# Structural/Functional Matches and Divergences of Phytoprostanes and Phytofurans with Bioactive Human Oxylipins

**DOI:** 10.3390/antiox7110165

**Published:** 2018-11-16

**Authors:** Sonia Medina, Ángel Gil-Izquierdo, Thierry Durand, Federico Ferreres, Raúl Domínguez-Perles

**Affiliations:** 1 CQM—Centro de Química da Madeira, Universidade da Madeira, Campus da Penteada, 9020-105 Funchal, Portugal; sonia.escudero@staff.uma.pt; 2 Research Group on Quality, Safety and Bioactivity of Plant Foods, Department of Food Science and Technology, CEBAS (CSIC), Campus University Espinardo, 30100 Murcia, Spain; federico@cebas.csic.es (F.F.); rdperles@cebas.csic.es (R.D.-P.); 3 Institut des Biomolécules Max Mousseron (IBMM), UMR 5247—CNRS, Faculty of Pharmacy, University of Montpellier—ENSCM, 34093 Montpellier, France; Thierry.Durand@umontpellier.fr

**Keywords:** plant oxylipins, mammals oxylipins, structural analogy, biological activity, SAR

## Abstract

Structure-activity relationship (SAR) constitutes a crucial topic to discover new bioactive molecules. This approach initiates with the comparison of a target candidate with a molecule or a collection of molecules and their attributed biological functions to shed some light in the details of one or more SARs and subsequently using that information to outline valuable application of the newly identified compounds. Thus, while the empiric knowledge of medicinal chemistry is critical to these tasks, the results retrieved upon dedicated experimental demonstration retrieved resorting to modern high throughput analytical approaches and techniques allow to overwhelm the constraints adduced so far to the successful accomplishment of such tasks. Therefore, the present work reviews critically the evidences reported to date on the occurrence of phytoprostanes and phytofurans in plant foods, and the information available on their bioavailability and biological activity, shedding some light on the expectation waken up due to their structural similarities with prostanoids and isoprostanes.

## 1. Introduction

In mammals, oxidative stress has been traditionally associated with the pathogenesis of several chronic diseases. This is caused by the high levels of reactive oxygen species (ROS) that, in mammals, are generated in mitochondria as a consequence of cellular breathing, and ROS production due to an increased metabolism. When the production of ROS surpass the reduction capacity of cells, this situation leads to oxidative damage of lipids, proteins, and nucleic acids, altering their functions [1]. Similarly, in plants, the oxidative reactions triggered by ROS are focused on α-linolenic acid (C18:3, n-3, ALA), the main polyunsaturated fatty acid (PUFA) in higher plants, giving rise to a broad spectrum of oxylipins [2]. In this respect, in 1998, Parchmann and Mueller described, for the first time, the production of phytoprostanes (PhytoPs), which are synthesized as a consequence of non-enzymatic lipid peroxidation initiated by the attack of ROS to ALA [3]. More recently, another series of plant oxylipins, phytofurans (PhytoFs), have been described [4]. These compounds are oxylipins sharing structural analogy with PhytoPs generated by non-enzymatic oxidative reactions as well, although higher oxygen pressures (>21%) tip the scale in favor of the PhytoFs synthesis [5]. These compounds are structurally similar to cis-(+)-12-oxo-phytodienoic acid (cis-OPDA) and induce a common set of genes that could be involved in the synthesis of jasmonic acid, with responsibilities in a number of pathways related to higher plants response to biotic and abiotic stress [6].

These compounds have been identified as components of the sensing and signaling system of higher plants, while their structural analogy to mammals oxylipins derived from arachidonic acid (C20:4, n-6, AA) (isoprostanes and prostaglandins), on which have been demonstrated a number of biological actions, have allowed to envisage PhytoPs not only as excellent biomarkers of the oxidative degradation in higher plants, but also biologically active molecules capable of mimicking the biological activity of prostanoids and isoprostanes in mammals after dietary ingestion [7,8].

The relevance of PhytoPs as lipid mediators has been established in recent years [9,10]. Hence, these previous findings have suggested new valuable biological properties for plant oxylipins in the frame of diverse pathophysiological situations and health constraints [11]. Some authors have also suggested that PhytoPs and PhytoFs may be generated in mammals from ALA due to its accumulation in specific tissues and cells, such as the adipose tissue and skin [12]. To date, the bioavailability of these compounds has been demonstrated in some extent, being described esterified circulating forms in plasma. In addition, the structural analogy of PhytoPs and PhytoFs to mammals’ prostanoids and isoprostanes has allowed proposing a common metabolic and excretion pathway by conjugation with glutathione, glucuronic acid, and/or sulfate [2,13,14].

The cumulative knowledge gathered in this regard suggests that the most frequent esterification of these compounds are to galactolipids in plants (*Arabidopsis thaliana*) and triglycerides [15], supporting that PhytoPs containing triglycerides may enter the intestine barrier in an intact form [13]. However, this argument has not been fully addressed to date, requiring the metabolism of PhytoPs and PhytoFs further elucidation.

The aim of this review is to focus a rational discussion regarding the evidences on the occurrence of PhytoPs and PhytoFs in plant foods, and the information available on their bioavailability and biological activity, shedding some light on the expectation waken up due to their structural similarities with prostanoids and isoprostanes.

## 2. Occurrence of Phytoprostanes and Phytofurans in Biological Systems. Physiological Significance

To establish the actual interest of these compounds as biologically active molecules responsible for physiological functions in higher plants and/or susceptible to be bioavailable once ingested by diet and to develop biological functions in vivo, firstly it is required to establish their occurrence in foods and foodstuffs (Table 1).

When analyzing the data reported in the literature on the profile and occurrence of PhytoPs and PhytoFs in the separate foods and food matrices, it is essential taking into consideration the evolution of the analytical instrumentation throughout the last years. Thus, regarding the detection methods used so far to identify and quantify PhytoPs and PhytoFs in plant matrices and complex biological systems, earlier reports have described different analytical approaches, such as gas chromatography coupled to mass spectrometry (GC–MS) [7,8,14,29,31], high-performance liquid chromatography (HPLC) coupled to a fluorescence detector [32], nuclear magnetic resonance of ^1^H or ^13^C [33], immunological approaches (Enzyme-Linked ImmunoSorbent Assay-ELISA) [34], and liquid chromatography coupled to mass spectrometry with tripe quadrupole technology (UHPLC-QqQ-MS/MS) [16,17,27]. Although the HPLC–UV analysis method is rapid, its sensitivity and specificity are not high enough to profile and quantify these compounds in complex matrices, especially in complex biological matrices, in which their concentration is frequently at the nanomolar, or even picomolar range. In this concern, so far, one of the most sensitive methods applied to the analysis of these compounds is GC–MS [29,32]. However, this analytical technology accounts with a critical drawback, the requirement of derivatization reactions [13]. This handicap can be overwhelmed by assessing plant materials and mammals’ biological samples on the occurrence of PhytoPs and PhytoFs by liquid chromatography coupled to mass spectrometry detection (LC/MS), because resorting these analytical techniques, derivatization is not needed. Indeed, to date, PhytoPs have only been measured using LC-MS technology. Thus, LC/MS has been demonstrated as more specific and sensitive because allowing their separation and identification of their respective stereoisomers, especially when applying UHPLC–QqQ-MS/MS technology [17].

In this sense, previous studies have reported the concentration of PhytoPs in a range of edible and non-edible plant materials, for instance in peppermint, birch, and tomato leaves, lime tree flowers, birch pollen, and valerian root. In addition, these plants oxylipins have also been detected in several plant cell cultures, such as *Nicotiana tabacum* (Solanaceae), *Glycine max* (Fabaceae), and *Arabidopsis thaliana* (Brassicaceae), among others [3,7,8,29]. As referred in Table 1 and Table 2, from the descriptions available to date, exceedingly high levels of free PhytoPs in birch pollen (32.44 × 10^3^ ng g^−1^ DW) have been noticed. Indeed, the analysis of the concentrations reported so far reveals two orders of magnitude higher than in most other fresh plant parts, which might be of significance for both plant physiology and the possible biological activity of PhytoPs in humans after dietary intake, intestinal absorption, and systemic spread.

### 2.1. Phytoprostanes and Phytofurans in Vegetable Oils

**Figure 1:**
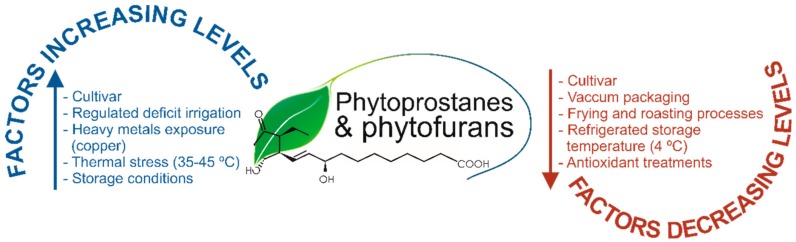
Factors modulating the oxylipins levels in plant foods.

In 2015, the PhytoPs profile and concentration were provided by the first time in commercial olive and sunflower oils, revealing that refined sunflower is featured by a profile and concentrations 20 and 8-fold higher than two types of olive oil: extra virgin olive oil (EVOO) and olive oil (OO), respectively [17]. The total content of PhytoPs reported were ~15.00, ~39.00, and ~297.00 ng mL^−1^ in EVOO, OO, and sunflower oils, respectively (Table 1). Given that these concentrations may not fit closely the concentration of ALA, the only precursor identified to date for these compounds, from these works it was suggested that the manufacture process could be the key for the differential PhytoP production, since most of the plant oils are subjected to a refining treatment, upon which further oxidation reactions may occur [17]. Hence, during these processes, it could go on in different extents the oxidative conditions that give rise the distinct concentration of PhytoPs. Moreover, in other study by the same authors, it was demonstrated the effect of regulated deficit irrigation (RDI) on the PhytoP content in *Olea europaea* L., cv. ‘Cornicabra’ EVOO [18] and reported that RDI during pit hardening critically influence the EVOO composition of free PhytoPs, also indicating a decisive influence of the genetic background (cultivar), oil extraction technology, and storage conditions prone to autoxidation [35] (Figure 1).

Continuing with oleaginous matrices, a very recent study assessed a wide range of vegetable oils, including EVOO, flax, sesame, argan, safflower seed, grapeseed, and palm oils on the content of PhytoPs and PhytoFs [16], pursuing to identify the most appropriate matrices to be eventually included in further dietary intervention studies devoted to establish the bioavailability of these compounds, in vivo. From this work, the authors retrieved that flax oil displays the highest concentration of both PhytoPs and PhytoFs described to date (119.150 × 10^3^ ng mL^−1^ and 37.92 × 10^3^ ng mL^−1^, respectively, Table 1), whereas palm and grapeseed oils were featured by concentrations that turn them as the most appropriate negative controls (<1.90 × 10^3^ ng mL^−1^ and <0.26 × 10^3^ ng mL^−1^, respectively). As mentioned before, authors identified these matrices as suitable to be also incorporated in in vitro assays designed to unravel mechanistic facts responsible for the biological activity demonstrated upon in vivo assays for PhytoPs and PhytoFs. In addition, it must be noticed that the PhytoP level was greater than the described by other authors in similar matrices [13], which cannot be explained exclusively by the analytical methodology applied (LC-MS vs. GC-MS), while the genetic features of the cultivars, the agro-climatic conditions to which the crops are exposed, or the technological settings used to obtain the edible oils, should also be taken into consideration. In this regard and according to the recent scientific literature, Figure 1 shows the most important factors contributing to the level of oxylipins in plant foods and foodstuffs.

Indeed, these factors may increase or decrease the oxylipins content, although, the response of plant oxylipins to changes in particular conditions, such as plant water status, was not as perceptible as expected, being required further studies that confirm their use as biomarkers of water stress, comparing the modification of the PhytoP level by water shortage with those of amino acids (proline and hydroxyproline, among others), already recognized as consistent markers of abiotic stress caused by irrigation deficit [18]. In the same way, table olive tree crop under RDI conditions during pit hardening and the processing of its fruits to obtain Spanish-style olives can be considered as complementary actions to enhance PhytoP content [19], and eventually also PhytoFs.

In addition to hydric stress, recently, it has been reported that thermal stress is a situation that modulates the content of PhytoPs and PhytoFs. In this regard, these compounds were quantified in melon leaves thermally stressed [30], showing that stressed melon plants grown covered with a plastic structure and exposed to high temperature (35–45 °C) were featured by 2.5-fold higher levels than non-stressed plants (control samples in open air conditions). Additionally, the assessment of several antioxidants treatments (salicylic acid, gallic acid, and *Ilex paraguariensis* extract) revealed a significant decrease of PhytoPs and PhytoFs in stressed samples treated with *I. paraguariensis* than in stressed samples without antioxidants. Hence, the consideration of these compounds as markers of oxidative stress (OS) in higher plants, would indicate that the use of natural antioxidants could reduce oxidative stress. In addition, from this work it can be extracted that PhytoFs also constitute useful biomarkers to monitor OS in plants, in addition to PhytoPs (more extensively demonstrated on this utility), providing additional tools to control OS under adverse environmental conditions and opening further research opportunities.

### 2.2. Phytoprostanes and Phytofurans in Nuts and Seeds

Another matrix that has been characterized regarding their content in PhytoPs and PhytoFs is almond kernel. In this case, it has been also identified the impact or irrigation deficit in the final concentration of these plants oxylipins. Indeed, a recent study demonstrated that nuts from rain-fed trees had lower individual and total PhytoP concentrations than those under irrigation [26]. This last study also quantified individual and total PhytoPs in 11 almond cultivars, under different agronomic conditions (conventional vs. ecological), and concluded that the PhytoPs profile varies greatly depending on the genotype (cultivars), where ‘Blanqueta’ showed the highest concentration (0.24 × 10^3^ ng g^−1^) and ‘Garriges’ was the almond cultivar featured by the lowest content (0.04 × 10^3^ ng g^−1^). In turn, the PhytoP profile of almonds is also affected by additional factors, such as the agricultural and irrigation system; however, some cultivars showed a higher total PhytoP content under conventional cultivation conditions, while others did so in the ecological system. This fact could be ascribed to the decisive effect of cultivar, agricultural system, and/or interaction between these two factors, which can make productions prone to autoxidation. Concerning the irrigation factor, the rain-fed almond trees displayed lower total PhytoP concentration than those under irrigation. Consequently, when these agronomic practices are applied, it could be expected an enhance of their content and, hence, an improved potential benefit on human health [26].

Additionally, in another recent study on the same matrix, the PhytoP profile in kernels of the almond cultivars ‘Colorada’, ‘Garriges’, ‘Largueta’, ‘Marcona’, and ‘Planeta’, fried or roasted and packaged under different conditions (air or vacuum), and exposed to storage temperatures of 5 and 24 °C, during 0, 3, or 6 months, and the type of processing (frying/roasting) was investigated. All these factors were demonstrated to have a significant impact on the PhytoPs profile of almonds, since the total PhytoP content was higher in the air atmosphere than in a vacuum system and most of the individual PhytoPs increased significantly during storage. However, interestingly, the storage time resulted in the synthesis of specific PhytoPs that were not detected at day 0 that allowed describing specific markers for quality control. In addition, the time was not the only factor affecting the concentration of PhytoPs during storage, but also temperature, producing the storage at 24 °C higher levels than 5 °C. Finally, upon this work was stated that the processing of almonds also influenced critically the level of PhytoPs since fried-salt and roasted almonds exhibited a decrease of the total content compared with the raw kernels [25]. The PhytoPs profile of three types of nuts (‘walnut’, ‘macadamia’, and ‘pecan’) processed by diverse frying conditions were also studied, revealing that the level of the separate compounds within plant oxylipins are influenced by both factors (cultivar and thermal stress) [27].

In 2015, for the first time, the presence of PhytoFs in nuts (pine nuts and walnuts) and seeds (flaxseed and chia seeds) was established [4], where the concentration of *ent*-16-(*RS*)-13-*epi*-ST-Δ^14^-9-PhytoF in walnuts and chia seeds was approximately 10- to 20-fold higher than in flaxseeds and pine nuts (Table 2). These recent studies reinforced the interest of PhytoPs and PhytoFs as biomarkers, not only to control the processing treatments that may influence the final food quality, but also to monitor the food authenticity, given the close relationship of the PhytoPs and PhytoFs profile and the specific matrix.

### 2.3. Phytoprostanes and Phytofurans in Rice

Regarding the effect of different cultivars on the PhytoP and PhytoF levels, a recent study provided further evidences on the relevance of the genetic background for the definition of the plant oxylipins status, by detecting and quantifying these compounds in 14 rice (*Oryza sativa* L.) cultivars, eight of the indica type and six of the japonica class [28]. Upon this work, it was revealed that total PhytoP content was significantly higher in japonica cultivars than in the indica ones. Moreover, the level of these compounds varied depending on the sample types from rice (bran > brown grain flours > white grain flours), as referred in Table 1. In addition, the concentration of PhytoFs detected in the samples was lower than the level of PhytoPs, this fact may be attributed to the specific physicochemical features of the matrices evaluated, with very low water content and lower oxygen pressure that does not give rise to the oxidation conditions required for the synthesis of phytoFs. Another important finding of this study was the positive and significant correlation between the total concentration of PhytoPs and PhytoFs. This information may be very useful for future research about new source of bioactive compounds with nutraceutical and pharmaceutical applications.

### 2.4. Phytoprostanes and Phytofurans in Musts and Wines

Another factor that may condition the level of plant oxylipins is the fermentation process. In this concern, one report about the PhytoP content of red wines and their respective musts (no fermented) was published in 2015 by Marhuenda and colleagues [11]. In this work, three different wines and musts were selected to investigate diverse winemaking processes and aging procedures (carbonic maceration wines/must (CMW/CMM), aged wines/musts (AW/AM), and high expression wines/musts (HEW/HEM). The grapes fruits to obtain HEW/HEM came from very old and low-yielding vineyards (>50 years old), which usually are exposed to more intense stressing factors than newer vineyards. Overall, the level of PhytoPs was identified as higher in wines samples than their respective musts in the case of CMW/CMM and AG/AM. However, in HEM samples larger amounts of PhytoPs than in HEW were detected, with the level of the D_1_-PhytoP 100-fold higher in the HEM in comparison with the other classes (Table 1). This may be due to differences in agronomic factors (vineyard age) could contribute to the different concentration of total PhytoPs, compared to HEM in the must from grapes grown in the new vineyards (CMM and AM). In addition, both vinification and aging (e.g., in oak barrels) may influence and change the initial PhytoP profile. Although, further studies are needed to elucidate the development of new compounds during wine production, this information may be of great relevance to farming, as well as for quality control during the winemaking process.

### 2.5. Phytoprostanes and Phytofurans in Macroalgae

Furthermore, the first study about of the naturally occurrence of PhytoPs in macroalgae was carried out by Barbosa and colleagues [20]. In this study, the PhytoP profile in 24 macroalgae species belonging to *Chlorophyta* (green algae), *Phaeophyta* (brown algae), and *Rhodophyta* (red algae) families was determined using UHPLC-QqQ-MS/MS technology. The total PhytoP levels ranged between 0.06 and 13.81 ng g^−1^ (Table 1), depending on the macroalgae species, where *Saccharina latissima*, which belongs to the *Phaeophyta phylum*, was identified as the macroalgae species with the highest amount of PhytoPs (13.81 ng g^−1^). However, no correlation between the amounts of ALA in algae samples and the PhytoP content was found, perhaps because of interspecies variability. This research suggested a new approach for the exploitation of marine organisms as valuable sources of oxylipins. More recently, PhytoPs and PhytoFs have been quantified in red and brown macroalgae by Micro-LC-MS/MS, where brown algae showed high level of the F_1_-PhytoP. Moreover, this assay was the first to detect and quantify PhytoFs in macroalgae (Table 2) [21]. These authors also investigated the effect of heavy metal (copper) exposure on the PhytoP and PhytoF levels, resulting in an increase of their concentration after 24 h of copper stress. Although more research is needed, the plant oxylipins could act as potential markers of marine and freshwater species contaminated by heavy metals.

### 2.6. Phytoprostanes and Phytofurans in Foodstuffs

In the food industry, food and foodstuffs processing can lead to one third of the production being discarded, resulting in high amounts of by-products (shells, seeds, leaves, and stones, among others) with limited uses. Several recent studies have been developed on the base of design new valorization alternatives to wastes from the agro-food industry, through the detection of tentatively bioactive PhytoPs in these by-products [22,23]. So, for instance, total phytoprostanes were quantified in shells from gulupa fruits (*Passiflora edulis* Sims f. *edulis*) revealing values ranging from 1.30 to 67.60 ng g^−1^ DW. In addition, equal determination in shells from banana passion fruits (*Passiflora tripartita* var. mollisima) and calyces from goldenberry (*Physalis peruviana*) allowed to report even higher concentrations (>21,000.00 ng g^−1^ DW) than other vegetable matrices previously studied, such as olives and almonds, among others (Table 1). Consequently, these studies provided new insights into the importance of fruit residues as a valuable option for the design of new products rich in these bioactive compounds.

The production of oxygenated fatty acids in plants is a characteristic response to pathogenesis, and frequently complemented by the release of reactive lipid products. In addition to their interest as indicators of biotic and abiotic stress, some plant oxylipins act as potent regulators, for instance, jasmonic acid and cyclopentenone lipids that are being suggested as capable to activate or repress gene expression upon the activation of conserved electrophilic groups [36]; however, this extent continue nowadays under explored and merits further research efforts.

## 3. What do We Know on the Bioavailability of Phytoprostanes and Phytofurans?

Nowadays, scarce data are available on application of animal or humans models regarding the bioavailability of PhytoPs and PhytoFs that are gathered in only two studies [13,14]. The gap on reliable information in this issue is mainly due to the difficulties of in vivo analysis related to their metabolism, since these compounds have been reported as relatively instable in vivo and rapidly excreted. Moreover, the analysis of oxylipins in general requires sophisticated techniques [17,37], that are being described in the last years, while standards for the identification and quantification of such analytes are synthesized by a limited number of research groups worldwide and not commercially available [4,5,38,39,40,41]. In this regard, the reduced number of standards available are pure, unesterified, compounds, while esterification occurring in nature remains a corner stone of the biology of these compounds that will require untargeted metabolomic approaches to be elucidated.

In the light of these works, the study of the dietary intake of plant oils (linseed, soy bean, olive, rapeseed, walnut, and grapeseed oils) by healthy (male) volunteers with regard to the PhytoPs metabolism revealed that these compounds are relatively resistant to the acidic pH of the stomach and could reach intact the small intestine. Upon this intervention, it was noticed that the ingestion of olive or soybean oils allow the intestinal absorption of the F_1_-PhytoPs. This circulates in plasma conjugated, even those esterification type remains to be clarified. Finally, the F_1_-PhytoP is excreted in free form by urine [14]. It is important to highlight that the limited number of compounds monitored (according to the availability of standards) allows to envisage that additional compounds belonging to this oxylipins family could be also absorbed at intestinal level. In addition, Barden and col. determined the plasma and urinary levels of F_1_-PhytoP after chronic consumption (four weeks) of olive oil and flax oil, containing 12.90 and 25.60 mg L^−1^, respectively [14]. The results showed higher plasma concentrations in those volunteers consuming flax oil (~5.9 nmol L^−1^), relatively to the ingestion of olive oil (~4.5 nmol L^−1^). The study by Barden and col. also suggested, with base on the correlation analysis developed, that the F_1_-PhytoP present in plasma were not only derived from its direct intestinal absorption, but also from the peroxidation, *in vivo*, of ALA, indicating that the final concentration of PhytoPs in humans cells and tissues depends on the concentration of both PhytoPs and ALA in plant foods [14].

Recently, 2,3,4,5,6,7-hexanor-16-F_1_-PhytoP has been suggested as a possible urinary metabolite produced by β-oxidation reactions [9]. In this respect, it is expected that the metabolism of PhytoPs and PhytoFs may be developed by the same route, previously described for isoprostanes in mammals. These, frequently, are not terminal products of the pathway and dehydrate in vivo to yield additional oxylipins because containing an α,β-unsaturated cyclopentenone ring system, which is a highly reactive electrophile substrate of the Michael reactions, forms adducts with cellular thiols, including those found on cysteine residues in proteins and glutathione. Hence, these cyclopentenone are metabolized *in vivo* by the action of the glutathione-*S*-transferase and uridine 5’-diphospho-glucuronyl transferase to form glutathione and glucuronide polar derivatives and excreted by urine [42]. Thus, as mentioned before, and given the structural similitudes of mammals and plats oxylipins it could be expected that PhytoPs and PhytoFs were metabolized and excreted by an equal route. Nevertheless, in peripheral blood these compounds have been found esterified to triglycerides and fatty acids, thus, it should be further explored a hypothetical participation of chylomicrons in the organic distribution and excretion of these compounds.

Although to date limited information is available on the metabolism of esterified PhytoPs and PhytoFs in vivo, and on the mechanisms responsible for the hydrolysis of these compounds towards free forms, such parallelisms could be extracted from the analysis of the reactions undergoing on mammals oxylipins. In this regard, it has been suggested, that minimal stimulus in the frame of complex cellular routes can be responsible for the activation of the activation of molecular mechanisms responsible for the hydrolysis of these compounds and thus, to their modulation of the general physiology of the involved cells. Between the mechanisms involved in these processes regarding eicosanoids, to date it has been identified the relevance of the kinase activation of enzymes or receptors and the oxidative inactivation of protein phosphatases [43].

However, even though it has been suggested the participation of PhytoPs and PhytoFs in the metabolic route upon which are processed prostanoids and isoprostanes, new research is required to confirm this fact, as well as to determine accurately the actual occurrence of such compounds in cells and tissues and the compounds and derivatives responsible for the separate biological activities that could be eventually identified.

Apart from the identification of the metabolic pathways involved in the turn-over of PhytoPs and PhytoFs in vivo, it is equally needed to gain a further insight in the additional factors that could affect the bioavailability of the compounds of interest, namely circadian rhythm, age, gender, gut microbiome or other people characteristics that may modulate the plant oxylipins metabolism and thus, their bioavailability and bioactivity. In this regard, to date it remains to be established the basic biochemical reactions that influence the concentration-activity relationship, which is essential to reach rational conclusions on the impact in health conditions of bioactive nutrients and non-nutrients, as is the case of PhytoPs and PhytoFs [43].

Likewise, to sort out the PhytoPs and PhytoFs bioactivity in vitro, it should be considered those tissues where ALA is accumulated, such as adipose tissue and skin to design assays with cell cultures. This is especially relevant because of PUFAs could be involved in an array of signaling pathways, providing as a result additional bioactive oxylipins responsible for the final biological activity, tentatively attributed to these compounds. So, in cells, in response to a given stimulus, it can activate distinct enzymes, such as phospholipase A2, which hydrolyze PUFAs allowing the release of unesterified compounds. These can interact with other cytosolic enzymes triggering the production of diverse bioactive oxylipins. These mechanisms that have been demonstrated regarding the production of eicosanoids (animal oxylipins) in cells [43], should be further explored for the occurrence of PhytoPs and PhytoFs, as well as the impact on the biological implications.

## 4. Comparison between Plant and Human Bioactive Oxylipins: Tentative Insights in the Structure-Activity Relationship

Concerning the biological interest of oxylipins, as mentioned before, these compounds have been broadly referred in the literature as extremely reliable markers of OS [8,29,44]. On the other hand, the evaluation of additional biological functions remains in its infancy, being mainly supported by comparisons between the compounds with similar chemical structure in plants and mammals with a weak experimental support. Anyway, and despite these efforts, to date, a gap of information remains on the specific structural attributes responsible for such bio-functions on plant and mammals oxylipins. Although given this situation, it is evident the requirement of additional data to further confirm the biological activity, some reports have compared the biological functions of structural analogs prostanoids and PhytoPs sharing biological actions, have provided much useful information [13,45,46,47].

To understand the equivalences between mammals and plant oxylipins, it is essential to review the biosynthesis of both classes of compounds. In respect to the reactions responsible for the synthesis of oxylipins, as mentioned before, the separate types share biochemical (non-enzymatic) mechanisms of fatty acids oxidation and cyclization. Upon this pathway, firstly is formed a H_2_-type cyclic fatty acid with a cyclopentenone endoperoxide from separate precursors (AA in mammals and ALA in higher plants). This is chemically unstable and gives rise to racemic isoprostanoids featured by prostaglandin D-, E-, and F-ring systems. From these, the D- and E-ring systems undergo further dehydration and isomerization to deoxy-J-ring isoprostanoids (D-ring class) and to form A- and B-ring isoprostanoids (E-ring class) [9]. These reactions are developed by mechanisms that are not well clarified, and may include exposure to acidity or alkalinity outside the typical physiological value around 7.4, for instance, the alkaline binding site on blood plasma albumin [48]. Thus, by these mechanisms, diverse structurally related classes are produced in mammals and plants in vivo [9], and from them, an array of varied (and tentatively matching) biological activities are expected as well.

Hence, interestingly, to identify the biological benefits and the modes of action of oxylipins, to date, a range of compounds (including mammals’ prostanoids and isoprostanes and plants’ PhytoPs and PhytoFs) have been synthesized and assessed. Nonetheless, these have been mainly used with the purpose of characterize the occurrence and modulation of their level of expression of such compounds under diverse pathophysiological conditions. In a lower extent, a number of mechanistic studies upon which it has been retrieved limited information on the biological activity have been also developed. Thus, between the biological activities identified, so far, immunomodulation, vasoconstriction, platelet activation and anti-aggregation, smooth muscle contraction of bronchi, and anti-inflammatory and apoptosis-inducing activities in different tissues can be noticed. In addition, isoprostanes have been suggested as mediators of oxidative stress in the pathophysiology of chronic cardiovascular, respiratory, and metabolic diseases [44].

### 4.1. Biological Functions of Phytoprostanes in Higher Plants

The biological potential of these compounds in mammals is also supported by their role in plant cells. In the frame of oxidative stress in plant cells, it has been proposed a close participation of mitogen-activated protein kinases (MAPK), which constitute key elements of the signal-transduction pathways. In this concern, it has been verified that the implication of MAPK could be induced by the dexoy-J_1_-PhytoP (both types I and II) [41]. Other cyclopentenone-based compounds, in this case represented by the A_1_- and B_1_-PhytoP, have been found competent to induce the expression of glutathione-*S*-transferase-1, an enzyme involved in the detoxification of lipid-peroxidation products, including the own PhytoPs and PhytoFs, as well as, for instance α,β-unsaturated aldehydes and ketones [29].

In this context, an array of studies carried out on various plant models have described PhytoPs as primordial mediators of the stress response in higher plants, inducing the synthesis of secondary anti-microbial metabolites, such as phytoalexins or antioxidants. These evidences suggest a tentative functionality as mediators of defense reactions, in response to oxidative stress in plants [32], as well as inhibiting cells growth and division [49], for which it is essential the regulation of the expression of genes related to detoxification processes, such as those codifying for proteins involved in the cytochrome-P450 system and heat-shock proteins (HSP70) [32,49].

### 4.2. Phytoprostanes as Immunomodulatory Molecules in Humans

**Figure 2:**
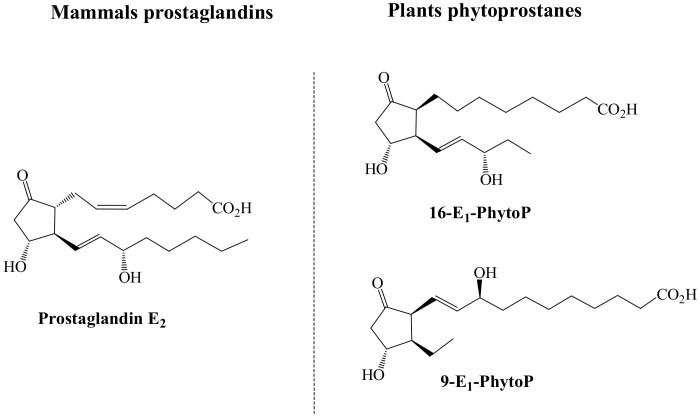
Chemical structure of PGE_2_ and E_1_-PhytoP sharing the capacity to polarize the immune response to TH2 pro-allergenic.

In humans, the first studies developed were focused on unraveling the capacity of PhytoPs to modulate cellular mechanisms involved in adaptive immunological responses [34,45,46,50]. In this regard, in the last decades it has been reported the immunomodulatory capacity of oxylipins by polarizing the type of CD4^+^-T-cells activated through the adaptive immune system to a type 2 helper (TH2) response (pro-allergenic) [46]. In the frame of this pathway, it has been described that E_1_-PhytoP is competent to mimic the biological activity of E_2_-prostaglandin (PGE_2_), which activity in vitro has been related with the inhibition of the production of IL-12 by dendritic (professional antigen presenting) cells, activating mechanisms dependent of the peroxisome proliferator activated receptor gamma (PPAR-γ) that lead the inhibition of the NF-kB activation [45,46]. Given the similarities between these two molecules (Figure 2), it has been suggested that the specific positions of the hydroxyl and keto groups on the cyclopentenone ring and their electrophilic features, could be responsible for the specific biological activity identified. Indeed, according to the molecules structures represented in Figure 2, it is suggested that the responsible for the mimicking functionality between E_1_-PhytoP and PGE_2_-is the responsibility of the 16-E_1_-PhytoP.

### 4.3. Anti-Inflammatory Capacity of Phytoprostanes in Humans

Apart from the immunomodulatory activity demonstrated, which seems to be a result of the structural analogy between PGE_2_-and 16-E_1_-PhytoP, prostaglandins and isoprostanes featured by the presence of an A-, J, or deoxy-J-ring system in their structure, have been confirmed on anti-inflammatory, pro-apoptotic activity on tumor cells, and antiviral properties in mammals, extensively reviewed by Straus et al. [47].

**Figure 3:**
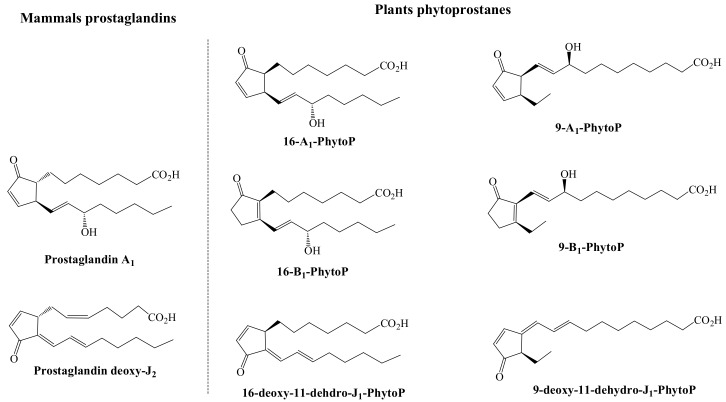
Chemical structure of the prostaglandin A_1_ and deoxy-J_2_ and the phytoprostanes A_1_, B_1_, and deoxy-J_1_ sharing the capacity to inhibit the NF-kB-mediated inflammatory response.

The anti-inflammatory properties associated to the presence of prostaglandins including into their structure of a cyclopentenone-ring system are developed in some extent by their capacity to impede the NF-kB-mediated signaling [47,51]. This functional property, jointly with the structural analogy between prostaglandins and PhytoPs, have prompted to assess plant cyclopentenone-based compounds on their capacity to mimic the anti-inflammatory activity of prostaglandins, by monitoring their capacity to inhibit the activation of the transcription factor NF-kB. For this, it was compared both series (9 and 16) of phytoprostanes A_1_, B_1_, and deoxy-J_1_ with the mammals’ PGA_1_ and deoxy-PGJ_2_ [13] (The chemical structure of these plant and mammals oxylipins is shown in Figure 3).

Upon this comparison, it was revealed that the A_1_-PhytoP and the two series of the deoxy-J_1_-PhytoP inhibited the transactivation of NF-kB in a dose-dependent manner, as the PGA_1_ and deoxy-PGJ_2_, respectively, although the operative concentrations of the deoxy-J_1_-PhytoP were even lower. On the other side, the 16-B_1_-PhytoP was inefficient or showed a much lower efficiency (9-B_1_-PhytoP) in displaying the inhibitory activity [13]. A further confirmation of the anti-inflammatory activity of these compounds was achieved upon monitoring the formation of nitric oxide, which revealed that even though A_1_-PhytoP and deoxy-J_1_-PhytoP are featured by a capacity comparable with that of the deoxy-PGJ_2_, being A_1_-PhytoP the most efficient inhibitor of the NO synthesis [13].

In addition, the study of the biological activity of PhytoPs has been extended to diverse cells types and tissues, including the nervous system. By applying this model, Minghetti and col. demonstrated that the B_1_-PhytoP displays neuroprotective activity on immature nerve cells (neuroblasts and oligodendrocytes) and promotes oligodendrocyte differentiation, via PPARγ activation [50]. This activity, even though it has not been previously described, seems to be developed by mimicking the mechanisms triggered by the deoxy-PGJ2 [10,13].

### 4.4. Phytoprostanes vs. Mammals Oxylipins Regarding Antitumor Activity

In addition to the anti-inflammatory activity of plants and mammals’ oxylipins, it has been described their capacity to induce apoptosis in an array of classes of malignant cells. Indeed, this capacity has been demonstrated on prostaglandins including cyclopentenone ring A- and J-system [52,53]. The comparison of these biological attributions with those of PhytoPs was developed by comparing their antitumor effect with the effect of the PGA_2_ and deoxy-PGJ_2_, revealing that 16-A_1_-PhytoP induces apoptosis of T-cell lymphoma in a higher extent than PGA_2_ (almost two-fold higher) but also than 9-A_1_-PhytoP. Interestingly, the 16-and 9-B_1_-PhytoP, structural analogs of the A_1_-PhytoP were found absolutely inactive [13]. In addition, these compounds inhibit malignant cells proliferation by modulating the expression of several genes related to the cell-cycle, codifying to chaperones heat shock proteins, transcription factors c-fos, Egr-1, and gadd153, the cyclin-dependent protein kinase inhibitor p21CIP1/WAF1, ubiquitin, protein disulfide isomerase, heme oxygenase, c-myc, N-myc, cyclin D1 and cdk4, and the insulin-like growth factor I, as is the case of the prostaglandin deoxy-J_2_ [47]. Again, according to the structural analogy it would be expected that the deoxy-J_1_-PhytoP could mimic this biological activity with different intensity.

Additionally, in a recent study, one of the few reporting the biological activity of PhytoFs, seven PhytoPs and one PhytoF were tested on breast cancer cell lines (MCF-7 and MDA-MB-231) to determine their effect on cell viability and metastatic capacity [54]. Upon this study, it was retrieved that the *ent*-9-L_1_-PhytoP lowers cell viability in both cell lines, while 16-F_1t_-PhytoP and 9-L_1_-PhytoP develop their inhibitory activity of MCF-7 and MDA-MB-231 cells, respectively. Regarding the influence of the referred cyclopentenone compounds on the metastatic capacity of the malignant cells, *ent*-9-(*RS*)-12-*epi*-ST-Δ^10^-13-PhytoF inhibited significantly MDA-MB-231 cell line migration, by enhancing the adhesion capacity of MDA-MB-231 cells [54].

These studies noticed the possible application of these compounds as adjuvant to improve the therapies available against breast cancer. This preliminary information should be further confirmed to retrieve more consistent conclusions on the mechanistic actions developed by PhytoPs and PhytoFs.

### 4.5. Key Structural Features and Mechanism of Action of Oxylipins for Biological Functions

Thus according to the information retrieved from the number of works available on the characterization of PhytoPs and PhytoFs, these plant oxylipins are featured by a cyclopentenone ring, exhibiting a chemical structure similar to prostaglandins and isoprostanes in mammals [2]. This seems to have a great influence on the biological properties of oxylipins. However, concerning plant PhytoPs, to date, this bioactive power has been only demonstrated in the frame of plant physiology and, in a much lower extent, regarding their immunomodulatory and anti-inflammatory capacity [7,34]. The cyclopentenone ring present as a common element in the chemical structure of both PhytoPs and PhytoFs and also in mammals’ prostaglandins and isoprostanes, constitutes an electrophilic site that provides to these compounds high reactivity with proteins [45]. In this regard, to date, it has been demonstrated that PhytoPs including in their structure the deoxy-J_1_ ring system are the most reactive electrophiles within the prostanoid series and readily bind by a Michael-type adduction reaction to free thiol groups in proteins, a molecular mechanism that has been proposed as essential in signal transduction [41]. However, additional research is required on their role in plant physiology and biological activity to understand the interaction between PhytoPs and PhytoFs with several higher plants regulating proteins.

From previous reports demonstrating that electrophilic cyclopentenone-based compounds, including cross-conjugated cyclopentenones of biological interest such as clavulone I, chlorovulone II, Chromomoric Acid C-II, TEI-9826, and the prostaglandin deoxy-J_2_ [55], it has been noticed that these compounds are competent to develop a range of biological activities [47]. This fact led foresaw that the presence of this chemical structure in PhytoPs and PhytoFs would allow them to develop equal actions by triggering similar mechanisms of action in humans, with diverse efficiency.

Regarding the mechanism of action, these biological functions have been proposed as a consequence of an array of interactions of the cyclopentenone electrophilic ring with diverse sub-units of target G-protein coupled prostanoids receptors, regarding to the prostaglandin E receptors (EP_1_, EP_2_, EP_3_, and EP_4_), the prostaglandin D receptor (DP), and the prostaglandin F receptor (FP), as well as to the nuclear receptor PPARγ [56,57,58]. In this regard, between the most relevant genes modified upon the activity of cyclopentenone molecules on PPARγ, those codifying to inducible nitric oxide synthase, tumor necrosis factor-α, gelatinase B, and cyclo-oxygenase-2 genes [59,60] have been noticed.

Interestingly, the specific biological functions of the separate oxylipins in mammals have been found a consequence, at least in part, of the degree of affinity by the currently known prostanoids receptors [47]. Thus, according to the information recently published by Ricciotti et al., the most relevant biological functions associated to each separate prostanoids receptors, which are unequally distributed in the diverse tissues and cell types, are triggered by the capacity of the ligands to induce and augment of intracellular cyclic adenosine monophosphate and the activation of the phosphatidylinositol metabolism, leading to the formation of inositol trisphosphate with the consequent mobilization of intracellular free calcium, and the activation of phospholipase C and guanine nucleotide exchange factor [61].

To understand the mechanisms of action of prostaglandins, which could be also triggered by plants’ PhytoPs and PhytoFs, it is essential to analyze their chemical structure. In this regard, prostaglandins including an A and J cyclopentenone ring system with an unsaturated carbonyl group and the presence of additional electrophilic carbons in the side chains, exhibit electrophilic centers that give rise more frequently to dienone compounds [62], and allows them to react with the cysteine residues of reduced glutathione or cellular proteins that seems to be responsible for a higher activity in a variety of assays [47]. The conjugation of cyclopentenone-based molecules to glutathione, for instance, could be developed spontaneously or catalyzed by the enzyme glutathione *S*-transferase, being more efficient the enzymatically mediated conjugations [63]. Interestingly, it has been demonstrated that the diverse types of glutathione *S*-transferase are characterized by stereo-selective metabolism reactions, according to differences in steric and electronic effects in mechanistic pathways. Thus, the conjugation of oxylipins to glutathione modulates, in some cases, the inhibition of the glutathione-S-transferase activity by binding covalently to cysteine residues in the active site of the enzyme [64].

Moreover, previous works have evidenced that a chemically reactive α,β-unsaturated carbonyl group is required for the successful development of a number of biological actions of cyclopentenone prostaglandins (and thus, tentatively of plants’ PhytoPs and PhytoFs as well). This demonstration is based on the fact that prostaglandins without cyclopentenone ring system are not competent to develop equal biological functions [65], while the conjugation of the reactive α,β-unsaturated carbonyl group with glutathione eliminates the activity observed on cyclopentenone prostaglandins [63,66,67]. These findings, jointly with the fact that cyclopentenone by itself has been demonstrated as capable to reproduce the biological activities of prostaglandins, reveal the relevance of the presence of this ring in the chemical structure for the accomplishment of the bio-functional roles [66,68,69].

In respect to the mechanism of action of these compounds, it has been suggested that they could induce the alkylation of cysteine residues of target proteins participating in key metabolic pathways in cells, due to the electrophile properties of the α,β-unsaturated carbonyl group. This would be responsible for the loss of function of the targets [65]. This fact has been further confirmed upon the study of the capacity of prostaglandins to inhibit NF-kB activity, in which participate two cysteine residues [69,70].

## 5. Conclusions and Future Prospects

In summary, cyclopentenone prostaglandins and isoprostanes containing an A- or deoxy-J-ring system are consistent with the development of an array of functions (immunomodulatory, anti-inflammatory, anti-viral, cytotoxic against malignant cells, and cytoprotective) in complex biological systems, and some of these activities can be developed by PhytoPs including into their chemical structure the A- and deoxy-J-ring system, in matching levels of concentration. Indeed, this fact further demonstrates that the functional capacity of both mammals and plants oxylipins in strongly driven by the specific structure of the cyclopentenone ring.

Given the limited information available on the Structure-Activity Relationship (SAR) of plant and mammals oxylipins, to retrieve rational prevision and conclusions on the newly identified molecules, the quantitative results obtained upon the computational perspective could be of great help. Therefore, according to the above referred information on the bioactivity of PhytoPs in plants, jointly with the presence of a cyclopentenone ring system in their structure, characteristic of molecules with demonstrated biological functions, these compounds are prone to develop valuable biological activities in vivo.

However, this fact requires a further “proof of concept” because even though the evident interest of gaining a further insight in the actual potential of plant oxylipins to display bio-functions, nowadays limited work has been developed to unravel this issue and to explore the SAR of oxylipins. Therefore, with this objective, it would be of much interest to apply enantiomerically pure PhytoPs obtained resorting to organic chemistry and, to date, used just to characterize plant foods and foodstuffs regarding the occurrence of PhytoPs and PhytoFs. The application of these pure compounds to diverse pathophysiological in vitro models, would contribute to provide SAR evidences, and pointing out the frame in which their biological activity would yield the most interesting benefits to health.

## Figures and Tables

**Table 1:** Natural occurrence of phytoprostanes in vegetables matrices.

Matrix/Plant Specie	Analytical Technique	Concentration [Reference]
Vegetable oils	UHPLC-QqQ-MS/MS	119,150 ng mL^−1^ (flax oil) [16]
		19,420 ng mL^−1^ (sesame/safflower oil) [16]
		16,310 ng mL^−1^ (EVOO) [16]
		<1900 ng mL^−1^ (argan, grapeseed, palm oil) [16]
	GC-MS	90–99,000 ng mL^−1^ (linseed, soy bean, olive, rapeseed, walnut and grapeseed oils) [13]
	UHPLC-QqQ-MS/MS	14.97 ng mL^−1^ (EVOO) [17]
		39.35 ng mL^−1^ (OO) [17]
		297.45 ng mL^−1^ (SO) [17]
	UHPLC-QqQ-MS/MS	31.92 ng mL^−1^ (EVOO from irrigated 100% trees) [18]
		67.87 ng mL^−1^ (EVOO from deficit-irrigated trees) [18]
Olives	UHPLC-QqQ-MS/MS	0.58–10 ng g^−1^ (raw flesh) [19]
		58–87 ng g^−1^ (treated flesh) [19]
Macroalgae	UHPLC-QqQ-MS/MS	0.06–13.81 ng g^−1^ DW [20]
Brown macroalgae	Micro-LC-MS/MS	310 ng g^−1^ FW (*Ectocarpus siliculosus*) [21]
		298.46 ng g^−1^ FW (*Laminaria digitata*) [21]
		78.51 ng g^−1^ FW (*Pelvetia canaliculata*) [21]
		28.91 ng g^−1^ FW (*Fucus spiralis*) [21]
Red macroalgae	Micro-LC-MS/MS	12.77 ng g^−1^ FW (*Osmundea pinnatifida*) [21]
		36.97 ng g^−1^ FW (*Grateloupia turuturu*) [21]
Passion fruits	UHPLC-QqQ-MS/MS	1.30–67.60 ng g^−1^ DW (*Passiflora edulis* Sims shell) [22]
		2.30–19,786 ng g^−1^ DW (*Passiflora tripartita* var. *mollisima* shell) [23]
		1.60–21,659.80 ng g^−1^ DW (*Physalis peruviana* calyx) [24]
Almond	UHPLC-QqQ-MS/MS	77 ng g^−1^ DW (raw Largueta cultivar) [25]
		9.30 ng g^−1^ DW (fried salt Largueta cultivar) [25]
		3.50 ng g^−1^ DW (roasted Largueta cultivar) [25]
		107 ng g^−1^ DW (raw Marcona cultivar) [25]
		18.85 ng g^−1^ DW (fried salt Marcona cultivar) [25]
		7.20 ng g^−1^ DW (roasted Marcona cultivar) [25]
		40–238 ng g^−1^ DW [26]
Nut kernels	UHPLC-QqQ-MS/MS	5 ng g^−1^ DW (raw walnut) [27]
		6 ng g^−1^ DW (raw Macadamia) [27]
		34 ng g^−1^ DW (fried Macadamia) [27]
		8 ng g^−1^ DW (raw Pecan) [27]
		19 ng g^−1^ DW (fried Pecan) [27]
Rice	UHPLC-QqQ-MS/MS	22.20–106.67 ng g^−1^ DW (rice bran) [28]
		10.14–22.53 ng g^−1^ DW (brown grain flour) [28]
		2.55–22.47 ng g^−1^ DW (white grain flour) [28]
*Betula pendula*	GC-MS	14.7 ng g^−1^ DW (leaves) [29]
*Nicotiana tabacum*	GC-MS	31.6 ng g^−1^ DW (cell culture) [3]
		53.2 ng g^−1^ DW (leaves) [29]
*Glycine max*	GC-MS	10.3 ng g^−1^ DW (cell culture) [29]
*Rauvolfia serpentina*	GC-MS	60.9 ng g^−1^ DW (cell culture) [3]
*Agrostis tenuis*	GC-MS	4.5 ng g^−1^ DW (cell culture) [3]
*Arabidopsis thaliana*	GC-MS	131.3 ng g^−1^ DW (leaves) [29]
*Lycopersicon esculentum*	GC-MS	16.9 ng g^−1^ DW (leaves) [29]
*Salix alba*	GC-MS	10.6 ng g^−1^ DW (leaves) [29]
*Rauvolfia serpentina*	GC-MS	20.5 ng g^−1^ DW (cell culture) [29]
Wine and must	UHPLC-QqQ-MS/MS	48.7 ng mL^−1^ (CMM) [11]
		20.4 ng mL^−1^ (AM) [11]
		430.9 ng mL^−1^ (HEM) [11]
		131.8 ng mL^−1^ (CMW) [11]
		213 ng mL^−1^ (AW) [11]
		199.8 ng mL^−1^ (HEW) [11]
*Cucumis melo* L.	Micro-HPLC-QTRAP	109–1146 ng g^−1^ (leaves) [30]
*Valeriana officinalis*	GC-MS	43 ng g^−1^ DW (root) [8]
*Mentha piperita*	GC-MS	76 ng g^−1^ DW (leaves) [8]
	GC-MS	76 ng g^−1^ DW (leaves) [7]
*Tilia cordata/platyphyllos*	GC-MS	135 ng g^−1^ DW (flowers) [8]
*Tilia cordata*	GC-MS	18.7 ng g^−1^ DW (leaves) [29]
*Hypericum perforatum*	GC-MS	211 ng g^−1^ DW [8]
*Betula pendula*/*pubescens*	GC-MS	1380 ng g^−1^ DW (leaves) [8]
		32,440 ng g^−1^ DW (pollen) [8]

AM: aged must; AW: aged wine; CMM: carbonic maceration must; CMW: carbonic maceration wine; DW: dry weight; EVOO: extra virgin olive oil; FW: fresh weight; GC-MS: gas chromatography mass spectrometry; HEM: high expression must; HEW: high expression wine; HPLC-QTRAP: high performance liquid chromatography- triple-quadrupole linear ion trap mass spectrometer; OO: olive oil; SO: sunflowers oil; UHPLC-QqQ-MS/MS: ultra-high performance liquid chromatography-triple quadrupole-tandem mass spectrometry.

**Table 2:** Natural occurrence of phytofurans in vegetables matrices.

Matrix/Plant Specie	Methodology	Concentration [Reference]
Vegetables oils	UHPLC-QqQ-MS/MS	37,920 ng mL^−1^ (Flax oil) [16]
		8850 ng mL^−1^ (EVOO) [16]
		30–260 ng mL^−1^ (sesame, safflower, argan, grapeseed, palm oil) [16]
Brown macroalgae	Micro-LC-MS/MS	490 ng g^−1^ FW (*Ectocarpus siliculosus*) [21]
		93.14 ng g^−1^ FW (*Laminaria digitata*) [21]
		70.03 ng g^−1^ FW (*Pelvetia canaliculata*) [21]
		41.14 ng g^−1^ FW (*Fucus spiralis*) [21]
Red macroalgae	Micro-LC-MS/MS	11.13 ng g^−1^ FW (*Osmundea pinnatifida*) [21]
		5.97 ng g^−1^ FW (*Grateloupia turuturu*) [21]
Rice	UHPLC-QqQ-MS/MS	1.59–27.74 ng g^−1^ DW (rice bran) [28]
		9.51–24.83 ng g^−1^ DW (brown grain flour) [28]
		0.06–1.79 ng g^−1^ DW (white grain flour) [28]
*Cucumis melo* L.	Micro-HPLC-QTRAP	130–4400 ng g^−1^ (leaves) [30]
Nuts and seeds	LC- MS/MS	0.30 ng g^−1^ DW (nuts) [4]
		0.70 ng g^−1^ DW (flax seed) [4]
		6.0 ng g^−1^ DW (chia seed) [4]
		9.0 ng g^−1^ DW (walnuts) [4]

DW: dry weight; EVOO: extra virgin olive oil; FW: fresh weight HPLC-QTRAP: high performance liquid chromatography- triple-quadrupole linear ion trap mass spectrometer; LC-MS/MS: liquid chromatography tandem mass spectrometry; UHPLC-QqQ-MS/MS: ultra-high performance liquid chromatography-triple quadrupole- tandem mass spectrometry.

## References

[1] Birben E., Sahiner U.M., Sackesen C., Erzurum S., Kalayci O. (2012). Oxidative stress and antioxidant defense. World Allergy Organ. J..

[2] Jacinta C.-G., Thierry D., Federico F., Sonia M., Arturo T., Ángel G.-I. (2015). Phytoprostanes. Lipid Technol..

[3] Parchmann S., Mueller M.J. (1998). Evidence for the Formation of Dinor Isoprostanes E1from α-Linolenic Acid in Plants. J. Biol. Chem..

[4] Cuyamendous C., Leung K.S., Durand T., Lee J.C., Oger C., Galano J.M. (2015). Synthesis and discovery of phytofurans: Metabolites of alpha-linolenic acid peroxidation. Chem. Commun..

[5] Cuyamendous C., Leung K.S., Bultel-Poncé V., Guy A., Durand T., Galano J.M., Lee J.C.Y., Oger C. (2017). Total synthesis and in vivo quantitation of phytofurans derived from α-linolenic acid. Eur. J. Org. Chem..

[6] Borrego E.J., Kolomiets M.V. (2016). Synthesis and Functions of Jasmonates in Maize. Plants.

[7] Imbusch R., Mueller M.J. (2000). Analysis of oxidative stress and wound-inducible dinor isoprostanes F1 (phytoprostanes F1) in plants. Plant Physiol..

[8] Imbusch R., Mueller M.J. (2000). Formation of isoprostane F2-like compounds (phytoprostanes F1) from α-linolenic acid in plants. Free Radic. Biol. Med..

[9] Galano J.M., Lee Y.Y., Oger C., Vigor C., Vercauteren J., Durand T., Giera M., Lee J.C. (2017). Isoprostanes, neuroprostanes and phytoprostanes: An overview of 25 years of research in chemistry and biology. Prog. Lipid Res..

[10] Minghetti L., Salvi R., Lavinia Salvatori M., Ajmone-Cat M.A., De Nuccio C., Visentin S., Bultel-Ponce V., Oger C., Guy A., Galano J.M. (2014). Nonenzymatic oxygenated metabolites of alpha-linolenic acid B1- and L1-phytoprostanes protect immature neurons from oxidant injury and promote differentiation of oligodendrocyte progenitors through PPAR-gamma activation. Free Radic. Biol. Med..

[11] Marhuenda J., Medina S., Díaz-Castro A., Martínez-Hernández P., Arina S., Zafrilla P., Mulero J., Oger C., Galano J.-M., Durand T. (2015). Dependency of Phytoprostane Fingerprints of Must and Wine on Viticulture and Enological Processes. J. Agric. Food Chem..

[12] Sinclair A.J., Attar-Bashi N.M., Li D. (2002). What is the role of alpha-linolenic acid for mammals?. Lipids.

[13] Karg K., Karg K., Dirsch V.M., Karg K., Dirsch V.M., Vollmar A.M., Cracowski J.-L., Laporte F., Mueller M.J. (2007). Biologically active oxidized lipids (phytoprostanes) in the plant diet and parenteral lipid nutrition. Free Radic. Res..

[14] Barden A.E., Croft K.D., Durand T., Guy A., Mueller M.J., Mori T.A. (2009). Flaxseed oil supplementation increases plasma F1-phytoprostanes in healthy men. J. Nutr..

[15] Ibrahim A., Schutz A.L., Galano J.M., Herrfurth C., Feussner K., Durand T., Brodhun F., Feussner I. (2011). The Alphabet of Galactolipids in Arabidopsis thaliana. Front. Plant Sci..

[16] Dominguez-Perles R., Abellan A., Leon D., Ferreres F., Guy A., Oger C., Galano J.M., Durand T., Gil-Izquierdo A. (2018). Sorting out the phytoprostane and phytofuran profile in vegetable oils. Food Res. Int..

[17] Collado-González J., Medina S., Durand T., Guy A., Galano J.-M., Torrecillas A., Ferreres F., Gil-Izquierdo A. (2015). New UHPLC–QqQ-MS/MS method for quantitative and qualitative determination of free phytoprostanes in foodstuffs of commercial olive and sunflower oils. Food Chem..

[18] Collado-González J., Pérez-López D., Memmi H., Gijón M.C., Medina S., Durand T., Guy A., Galano J.M., Fernández D.J., Carro F. (2016). Effect of the season on the free phytoprostane content in Cornicabra extra virgin olive oil from deficit-irrigated olive trees. J. Sci. Food Agric..

[19] Collado-González J., Moriana A., Girón I.F., Corell M., Medina S., Durand T., Guy A., Galano J.-M., Valero E., Garrigues T. (2015). The phytoprostane content in green table olives is influenced by Spanish-style processing and regulated deficit irrigation. LWT-Food Sci. Technol..

[20] Barbosa M., Collado-Gonzalez J., Andrade P.B., Ferreres F., Valentao P., Galano J.M., Durand T., Gil-Izquierdo A. (2015). Nonenzymatic alpha-Linolenic Acid Derivatives from the Sea: Macroalgae as Novel Sources of Phytoprostanes. J. Agric. Food Chem..

[21] Vigor C., Reversat G., Rocher A., Oger C., Galano J.-M., Vercauteren J., Durand T., Tonon T., Leblanc C., Potin P. (2018). Isoprostanoids quantitative profiling of marine red and brown macroalgae. Food Chem..

[22] Medina S., Collado-González J., Ferreres F., Londoño-Londoño J., Jiménez-Cartagena C., Guy A., Durand T., Galano J.-M., Gil-Izquierdo A. (2017). Quantification of phytoprostanes–bioactive oxylipins–and phenolic compounds of Passiflora edulis Sims shell using UHPLC-QqQ-MS/MS and LC-IT-DAD-MS/MS. Food Chem..

[23] Medina S., Collado J., Ferreres F., Londoño J., Jiménez-Cartagena C., Guy A., Durand T., Galano J.-M., Gil-Izquierdo A. (2017). Valorization strategy of banana passion fruit shell wastes: An innovative source of phytoprostanes and phenolic compounds and their potential use in pharmaceutical and cosmetic industries. J. Food Nutr. Res..

[24] Medina S., Collado-González J., Ferreres F., Londoño-Londoño J., Jiménez-Cartagena C., Guy A., Durand T., Galano J.M., Gil-Izquierdo A. (2018). Potential of Physalis peruviana calyces as a low-cost valuable resource of phytoprostanes and phenolic compounds. J. Sci. Food Agric..

[25] Carrasco-Del Amor A.M., Aguayo E., Collado-González J., Guy A., Galano J.-M., Durand T., Gil-Izquierdo Á. (2016). Impact of packaging atmosphere, storage and processing conditions on the generation of phytoprostanes as quality processing compounds in almond kernels. Food Chem..

[26] Carrasco-Del Amor A., Collado-Gonzalez J., Aguayo E., Guy A., Galano J., Durand T., Gil-Izquierdo A. (2015). Phytoprostanes in almonds: Identification, quantification, and impact of cultivar and type of cultivation. RSC Adv..

[27] Carrasco-Del Amor A.M., Aguayo E., Collado-González J., Guy A., Galano J.-M., Durand T., Gil-Izquierdo Á. (2017). Impact of processing conditions on the phytoprostanes profile of three types of nut kernels. Free Radic. Res..

[28] Pinciroli M., Domínguez-Perles R., Abellán A., Guy A., Durand T., Oger C., Galano J.M., Ferreres F., Gil-Izquierdo A. (2017). Comparative Study of the Phytoprostane and Phytofuran Content of indica and japonica Rice (*Oryza sativa* L.) Flours. J. Agric. Food Chem..

[29] Thoma I., Loeffler C., Sinha A.K., Gupta M., Krischke M., Steffan B., Roitsch T., Mueller M.J. (2003). Cyclopentenone isoprostanes induced by reactive oxygen species trigger defense gene activation and phytoalexin accumulation in plants. Plant J. Cell Mol. Biol..

[30] Yonny M.E., Rodríguez Torresi A., Cuyamendous C., Réversat G., Oger C., Galano J.-M., Durand T., Vigor C., Nazareno M.A. (2016). Thermal Stress in Melon Plants: Phytoprostanes and Phytofurans as Oxidative Stress Biomarkers and the Effect of Antioxidant Supplementation. J. Agric. Food Chem..

[31] Thoma I., Krischke M., Loeffler C., Mueller M.J. (2004). The isoprostanoid pathway in plants. Chem. Phys. Lipids.

[32] Loeffler C., Berger S., Guy A., Durand T., Bringmann G., Dreyer M., von Rad U., Durner J., Mueller M.J. (2005). B(1)-Phytoprostanes Trigger Plant Defense and Detoxification Responses. Plant Physiol..

[33] Vázquez-Romero A., Verdaguer X., Riera A. (2009). General approach to prostanes b1 by intermolecular pauson–khand reaction: Syntheses of methyl esters of prostaglandin b1 and phytoprostanes 16-b1-phytop and 9-l1-phytop. Eur. J. Org. Chem..

[34] Gutermuth J., Bewersdorff M., Traidl-Hoffmann C., Ring J., Mueller M.J., Behrendt H., Jakob T. (2007). Immunomodulatory effects of aqueous birch pollen extracts and phytoprostanes on primary immune responses in vivo. J. Allergy Clin. Immunol..

[35] Collado-Gonzalez J., Perez-Lopez D., Memmi H., Gijon M.C., Medina S., Durand T., Guy A., Galano J.M., Ferreres F., Torrecillas A. (2015). Water deficit during pit hardening enhances phytoprostanes content, a plant biomarker of oxidative stress, in extra virgin olive oil. J. Agric. Food Chem..

[36] Farmer E.E., Almeras E., Krishnamurthy V. (2003). Jasmonates and related oxylipins in plant responses to pathogenesis and herbivory. Curr. Opin. Plant Biol..

[37] Vigor C., Bertrand-Michel J., Pinot E., Oger C., Vercauteren J., Le Faouder P., Galano J.-M., Lee J.C.-Y., Durand T. (2014). Non-enzymatic lipid oxidation products in biological systems: Assessment of the metabolites from polyunsaturated fatty acids. J. Chromatogr. B.

[38] Cuyamendous C., de la Torre A., Lee Y.Y., Leung K.S., Guy A., Bultel-Ponce V., Galano J.M., Lee J.C., Oger C., Durand T. (2016). The novelty of phytofurans, isofurans, dihomo-isofurans and neurofurans: Discovery, synthesis and potential application. Biochimie.

[39] El Fangour S., Guy A., Despres V., Vidal J.P., Rossi J.C., Durand T. (2004). Total synthesis of the eight diastereomers of the syn-anti-syn phytoprostanes F1 types I and II. J. Org. Chem..

[40] El Fangour S., Guy A., Vidal J.P., Rossi J.C., Durand T. (2005). A flexible synthesis of the phytoprostanes B1 type I and II. J. Org. Chem..

[41] Iqbal M., Evans P., Lledo A., Verdaguer X., Pericas M.A., Riera A., Loeffler C., Sinha A.K., Mueller M.J. (2005). Total synthesis and biological activity of 13,14-dehydro-12-oxo-phytodienoic acids (deoxy-J1-phytoprostanes). ChemBioChem.

[42] Milne G.L., Yin H., Morrow J.D. (2008). Human biochemistry of the isoprostane pathway. J. Biol. Chem..

[43] Lands B. (2017). Highly unsaturated fatty acids (HUFA) mediate and monitor food’s impact on health. Prostaglandins Other Lipid Mediat..

[44] Montuschi P., Barnes P.J., Roberts L.J. (2004). Isoprostanes: Markers and mediators of oxidative stress. FASEB J..

[45] Gilles S., Mariani V., Bryce M., Mueller M.J., Ring J., Jakob T., Pastore S., Behrendt H., Traidl-Hoffmann C. (2009). Pollen-Derived E1-Phytoprostanes Signal via PPAR-γ and NF-κB-Dependent Mechanisms. J. Immunol..

[46] Traidl-Hoffmann C., Mariani V., Hochrein H., Karg K., Wagner H., Ring J., Mueller M.J., Jakob T., Behrendt H. (2005). Pollen-associated phytoprostanes inhibit dendritic cell interleukin-12 production and augment T helper type 2 cell polarization. J. Exp. Med..

[47] Straus D.S., Glass C.K. (2001). Cyclopentenone prostaglandins: New insights on biological activities and cellular targets. Med. Res. Rev..

[48] Fitzpatrick F.A., Wynalda M.A. (1981). Albumin-lipid interactions: Prostaglandin stability as a probe for characterizing binding sites on vertebrate albumins. Biochemistry.

[49] Mueller S., Hilbert B., Dueckershoff K., Roitsch T., Krischke M., Mueller M.J., Berger S. (2008). General detoxification and stress responses are mediated by oxidized lipids through TGA transcription factors in Arabidopsis. Plant Cell.

[50] Mariani V., Gilles S., Jakob T., Thiel M., Mueller M.J., Ring J., Behrendt H., Traidl-Hoffmann C. (2007). Immunomodulatory mediators from pollen enhance the migratory capacity of dendritic cells and license them for Th2 attraction. J. Immunol..

[51] Musiek E.S., Gao L., Milne G.L., Han W., Everhart M.B., Wang D., Backlund M.G., DuBois R.N., Zanoni G., Vidari G. (2005). Cyclopentenone isoprostanes inhibit the inflammatory response in macrophages. J. Biol. Chem..

[52] Kondo M., Shibata T., Kumagai T., Osawa T., Shibata N., Kobayashi M., Sasaki S., Iwata M., Noguchi N., Uchida K. (2002). 15-Deoxy-Δ(12,14)-prostaglandin J(2): The endogenous electrophile that induces neuronal apoptosis. Proc. Natl. Acad. Sci. USA.

[53] Zernecke A., Erl W., Fraemohs L., Lietz M., Weber C. (2003). Suppression of endothelial adhesion molecule up-regulation with cyclopentenone prostaglandins is dissociated from IkappaB-alpha kinase inhibition and cell death induction. FASEB J..

[54] Gutierrez-Pajares J., Oger C., Galano J.M., Durand T., Chevalier S., Frank P. (2016). 627-Oxidized products of α-linolenic acid negatively affect cell survival and motility of breast cancer cells. Eur. J. Cancer.

[55] Iqbal M., Evans P. (2003). Conjugate addition–Peterson olefination reactions: Expedient routes to cross conjugated dienones. Tetrahedron Lett..

[56] Forman B.M., Tontonoz P., Chen J., Brun R.P., Spiegelman B.M., Evans R.M. (1995). 15-Deoxy-delta 12, 14-prostaglandin J2 is a ligand for the adipocyte determination factor PPAR gamma. Cell.

[57] Kliewer S.A., Lenhard J.M., Willson T.M., Patel I., Morris D.C., Lehmann J.M. (1995). A prostaglandin J2 metabolite binds peroxisome proliferator-activated receptor gamma and promotes adipocyte differentiation. Cell.

[58] Narumiya S., Sugimoto Y., Ushikubi F. (1999). Prostanoid receptors: Structures, properties, and functions. Physiol. Rev..

[59] Jiang C., Ting A.T., Seed B. (1998). PPAR-gamma agonists inhibit production of monocyte inflammatory cytokines. Nature.

[60] Ricote M., Li A.C., Willson T.M., Kelly C.J., Glass C.K. (1998). The peroxisome proliferator-activated receptor-gamma is a negative regulator of macrophage activation. Nature.

[61] Ricciotti E., FitzGerald G.A. (2011). Prostaglandins and inflammation. Arterioscler. Thromb. Vasc. Biol..

[62] Noyori R., Suzuki M. (1993). Organic synthesis of prostaglandins: Advancing biology. Science.

[63] Atsmon J., Sweetman B.J., Baertschi S.W., Harris T.M., Roberts L.J. (1990). Formation of thiol conjugates of 9-deoxy-D9 D12(E)-prostaglandin D2 and D12(E)-prostaglandin D2. Biochemistry.

[64] Van Iersel M.L., Cnubben N.H., Smink N., Koeman J.H., van Bladeren P.J. (1999). Interactions of prostaglandin A2 with the glutathione-mediated biotransformation system. Biochem. Pharmacol..

[65] Fukushima M. (1990). Prostaglandin J2-anti-tumour and anti-viral activities and the mechanisms involved. Eicosanoids.

[66] Bui T., Straus D.S. (1998). Effects of cyclopentenone prostaglandins and related compounds on insulin-like growth factor-I and Waf1 gene expression. Biochim. Biophys. Acta.

[67] Kim D.H., Kim J.H., Kim E.H., Na H.K., Cha Y.N., Chung J.H., Surh Y.J. (2009). 15-Deoxy-Delta12,14-prostaglandin J2 upregulates the expression of heme oxygenase-1 and subsequently matrix metalloproteinase-1 in human breast cancer cells: Possible roles of iron and ROS. Carcinogenesis.

[68] Rossi A., Elia G., Santoro M.G. (1996). 2-Cyclopenten-1-one, a new inducer of heat shock protein 70 with antiviral activity. J. Biol. Chem..

[69] Straus D.S., Pascual G., Li M., Welch J.S., Ricote M., Hsiang C.-H., Sengchanthalangsy L.L., Ghosh G., Glass C.K. (2000). 15-Deoxy-Δ(12,14)-prostaglandin J(2) inhibits multiple steps in the NF-κB signaling pathway. Proc. Natl. Acad. Sci. USA.

[70] Rossi A., Kapahi P., Natoli G., Takahashi T., Chen Y., Karin M., Santoro M.G. (2000). Anti-inflammatory cyclopentenone prostaglandins are direct inhibitors of IkappaB kinase. Nature.

